# Wild Sorghum as a Promising Resource for Crop Improvement

**DOI:** 10.3389/fpls.2020.01108

**Published:** 2020-07-17

**Authors:** Galaihalage K. S. Ananda, Harry Myrans, Sally L. Norton, Roslyn Gleadow, Agnelo Furtado, Robert J. Henry

**Affiliations:** ^1^ Queensland Alliance for Agriculture and Food Innovation, The University of Queensland, St Lucia, QLD, Australia; ^2^ School of Biological Sciences, Monash University, Clayton, VIC, Australia; ^3^ Australian Grains Genebank, Agriculture Victoria, Horsham, VIC, Australia

**Keywords:** sorghum, crop wild relatives, crop improvement, cyanogenesis, wild sorghum

## Abstract

*Sorghum bicolor* (L.) Moench is a multipurpose food crop which is ranked among the top five cereal crops in the world, and is used as a source of food, fodder, feed, and fuel. The genus *Sorghum* consists of 24 diverse species. Cultivated sorghum was derived from the wild progenitor *S. bicolor* subsp. *verticilliflorum*, which is commonly distributed in Africa. Archeological evidence has identified regions in Sudan, Ethiopia, and West Africa as centers of origin of sorghum, with evidence for more than one domestication event. The taxonomy of the genus is not fully resolved, with alternative classifications that should be resolved by further molecular analysis. Sorghum can withstand severe droughts which makes it suitable to grow in regions where other major crops cannot be grown. Wild relatives of many crops have played significant roles as genetic resources for crop improvement. Although there have been many studies of domesticated sorghum, few studies have reported on its wild relatives. In *Sorghum*, some species are widely distributed while others are very restricted. Of the 17 native sorghum species found in Australia, none have been cultivated. Isolation of these wild species from domesticated crops makes them a highly valuable system for studying the evolution of adaptive traits such as biotic and abiotic stress tolerance. The diversity of the genus *Sorghum* has probably arisen as a result of the extensive variability of the habitats over which they are distributed. The wild gene pool of sorghum may, therefore, harbor many useful genes for abiotic and biotic stress tolerance. While there are many examples of successful examples of introgression of novel alleles from the wild relatives of other species from Poaceae, such as rice, wheat, maize, and sugarcane, studies of introgression from wild sorghum are limited. An improved understanding of wild sorghums will better allow us to exploit this previously underutilized gene pool for the production of more resilient crops.

## Introduction

*Sorghum bicolor* (L.) Moench, commonly known as sorghum, is ranked among the five main cereal crops in the world (Mace et al., 2009; Venkateswaran et al., 2014). It plays a vital role in global food production and is the staple food of billions of people (Mace et al., 2009). Sorghum is a multipurpose crop cultivated for grain, sweet stem, forage, and broomcorn. It also serves as a source of fuel, bioethanol, alcoholic beverages, and building materials. It is one of the most important food crops of arid and semi-arid regions of the world, whereas in developed countries it is grown mainly for forage and animal feed (Hariprasanna and Patil, 2015; Venkateswaran et al., 2019a). Currently, the USA has the world’s greatest total sorghum production, followed by Nigeria, India, and Mexico, with an average global production of 50 megatons per year (FAOSTAT, 2019). Sorghum is well adapted to high temperature, dry conditions and it is surprising that it is not even more widely grown. The lower global production of sorghum, relative to other cereals such as wheat and rice, might be increased by the exploitation of the hitherto untapped potential of the extensive gene pool of crop wild relatives (CWR) in the genus (Sasaki and Antonio, 2009). The use of *Sorghum* genetic resources is most immediately applicable to production of improved sorghum varieties. *Sorghum* is a genus within the tribe, Andropogoneae that includes other genera of plants such as *Saccharum* (Bonnett and Henry, 2011) and *Miscanthus* (Anzoua et al., 2011) that are important biomass crops. Sugarcane (*Saccharum*) and sorghum (*Sorghum*) are closely related and may be inter-crossed (Gupta et al., 1978). CWR in the *Sorghum* genus may also be a genetic resource to support the development of new crops across the tribe either by introgression of useful genes into genera such as *Saccharum* or by domestication of further *Sorghum* species (Dillon et al., 2007c).

CWR are plant species that are closely related to a domesticated crop, from anywhere in the world, including crop progenitors, landraces, and closely related taxa not historically involved in agriculture. They represent one of the key sources of new genetic material to introduce to crop lines through traditional breeding and, to a lesser extent, genetically modified (GM) crops. The use of CWR by agricultural scientists started to become a regular practice in the 1940s (Meilleur and Hodgkin, 2004). They have since been used to produce new lines of many globally important crops, improving traits such as disease and pest resistance, nutritional value, yield, and tolerance to abiotic stresses in crops such as wheat, tomatoes, rice, and many others (Prescott-Allen and Prescott-Allen, 1986; Hajjar and Hodgkin, 2007). CWR are seen by many as an invaluable source of diversity which should be drawn upon to further enhance crops in terms of commercial value, and to facilitate adaptation to changing environments and pathogens (Hoyt, 1988; Jarvis et al., 2008; Dempewolf et al., 2014; Brozynska et al., 2016). In monetary terms, it is estimated that the genetic resources they are worth over US$150bn (Tyack et al., 2015), highlighting the vital role they could potentially play in agriculture. Here, we refer to sorghum’s CWR as the wild taxa in the genus *Sorghum* Moench, including sorghum’s progenitors, but not landraces. Species names and ranks were standardized according to the USDA (2020). This review aims to understand the historical and current uses of sorghum crops and difficulties facing sorghum agriculture, and explores CWR’s potential as viable resources for future genetic improvement of the crop. To do this we discuss the origins and domestication of the crop, summarize and clarify what is known of the taxonomy of the genus and the phylogenetic relationships between subgenera, the barriers to gene flow and the potential for crop improvement.

## Origins and Distribution of Domesticated Sorghum

The earliest evidence of use of wild sorghum as a food is from the Sahara, around 7500 BC, where hunter-gatherers lived (Venkateswaran et al., 2019a). Similarly, a recent study by Winchell et al. (2017), has shown that the earliest domesticated sorghums are found in Neolithic populations of Sudan around fourth millennium BC. The exact origin and location of sorghum domestication is debated (De Wet et al., 1970; Venkateswaran et al., 2019a), however, archaeological evidence supports domestication in eastern Sudan around 3000 BC (Fuller and Stevens, 2018). Some studies suggest that there may have been more than one domestication event, potentially explaining the origin of the group *guinea-margaritiferum* of genus *Sorghum*, which was domesticated more recently (Kimber, 2000; Mace et al., 2013). According to archaeological evidence, *S. bicolor* originated from its wild progenitor *Sorghum bicolor* (L.) Moench subsp. *verticilliflorum* (Steud.) de Wet ex Wiersema & J. Dahlb., which is commonly distributed in Africa (De Wet and Harlan, 1971; De Wet, 1978; Doggett, 1988). There is no direct evidence available to suggest any contribution of other wild relatives viz., *Sorghum propinquum* (Kunth) Hitchc. and *Sorghum halepense* (L.) Pers. to cultivated sorghum, as suggested by Doggett (1988). Rowley-Conwy et al. (1997), proposed three hypotheses for sorghum domestication. The first hypothesis is based on the studies of Murdock (1959), which described an independent nuclear Mande center in West Africa. The next hypothesis is that the origin of sorghum could be in eastern Sahara, around 9700-6200 BC (Ehret, 2014), and the final hypothesis relies on the evidence of the race *durra* in India back in 4000 BC.

From its first ancestor in Africa, domesticated sorghum was distributed across the globe by various means—most commonly along trade routes. From East Africa, cultivated sorghum was moved across eastern and southern Africa as a result of human migration (Mann et al., 1983). It was then introduced to India *via* the Middle East trade routes (Mann et al., 1983). Doggett (1988), reported overland routes from East Africa and Somalia *via* Aden. The earliest *Sorghum* species found in India was *S. bicolor* and evidence for domestication and cultivation dating back to c.2000–1700 BC was found in the Indus Valley (Meadow, 1996; Fuller, 2003). Since then, sorghum has played a key role in agriculture in India (Kleih et al., 2000) and India is now considered to be its secondary center of diversity (Appa et al., 1996).

Sorghum was introduced to China from India, again *via* sea and overland trade routes. There are several hypotheses on how sorghum arrived in China. One of the possible ways was through the river valleys of Indochina (Venkateswaran et al., 2019a). However, Hagerty (1941) claims that the emperor Genghis Khan introduced sorghum to China after his voyage to South Asia between AD 1206–1228. The *Amber cane sorgos* are related to eastern African sorghums whereas the *Kaoliangs* probably originated from the *Sorghum* *bicolor* introduced from India (Doggett, 1988). There is evidence that *Kaoliangs* might be derived from native wild diploid sorghum (Harlan, 1995). The Yellow River Valley is considered to be the area where the earliest sorghum was cultivated based on archaeological evidence (Venkateswaran et al., 2019a). From China, sorghum was brought to the USA by the slave traders in the 19^th^ century. According to Martin (1936), the first sorghum to be introduced to the USA was the Chinese *Amber* in 1853. Sorghum was introduced to Queensland, Australia, in the 1900s by Americans (Venkateswaran et al., 2014). Since then sorghum has become a major summer crop in Australia, accounting for 5% of the global export of sorghum globally (Venkateswaran et al., 2019a).

## Taxonomy of *Sorghum*

**Figure 1:**
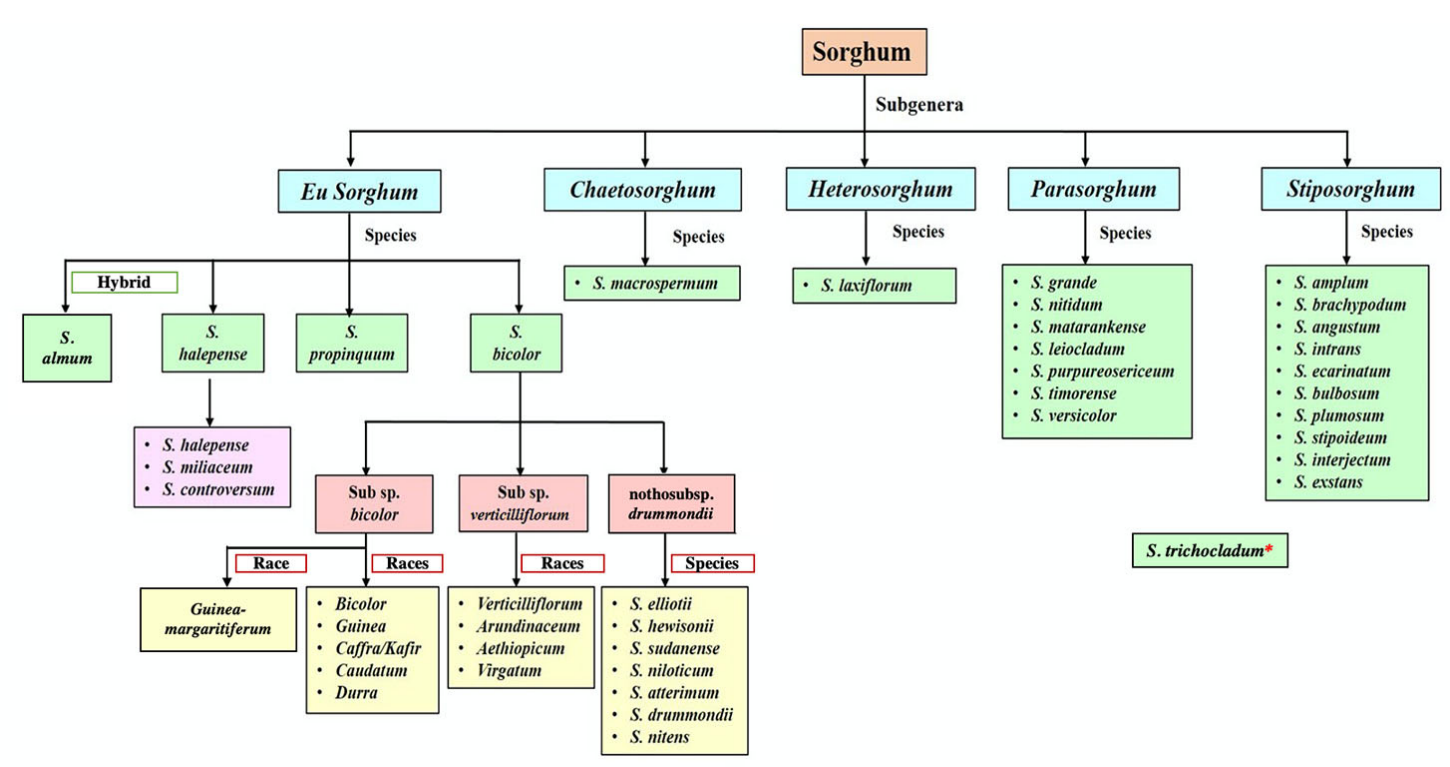
Classification of *Sorghum* (De Wet, 1978; Dillon et al., 2007a; Wiersema and Dahlberg, 2007; Venkateswaran et al., 2019a; USDA, 2020). *The exact position within the phylogeny is still uncertain.

The genus *Sorghum* was first classified as *Holcus* by Linnaeus in 1753 and constituted three species; *Holcus* *sorghum*, *Holcus* *saccaratus* and *Holcus* *tricolor*. *Sorghum* was separated out from the genus *Holcus* by Moench in 1794 (Venkateswaran et al., 2019a). Following these classifications, domesticated sorghum was formally recognized as *Sorghum bicolor* (L.) Moench (Venkateswaran et al., 2014; Hariprasanna and Patil, 2015). According to the current classification, sorghum belongs to the kingdom Plantae, division Magnoliophyta, class Liliopsida, order Cyperales, family Poaceae, tribe Andropogoneae, subtribe Sorghinae, and genus *Sorghum* (Hariprasanna and Patil, 2015). In Snowden’s classification, sorghum was divided into two main sections, *Eu-sorghum* and *Parasorghum*, based on morphological traits such as color of grains and glumes and persistence of pedicellate spikelets (Snowden, 1955). However, five subgenera of *Sorghum* are now recognized: *Eu-sorghum*, *Chaetosorghum*, *Heterosorghum*, *Parasorghum*, and *Stiposorghum* (Garber and Snyder, 1951; Harlan and de Wet, 1972; De Wet, 1978; Lazarides et al., 1991), based on morphological characters (**Figure 1**). Despite *S. bicolor* having been domesticated in East Africa, 17 Sorghum species are native to Australia. Of these, 13 are endemic, emphasizing the need to preserve sorghum’s CWR nationally. Native Australian species are present in every *Sorghum* subgenus, excepting *Eu-sorghum*.

The exact number of species in this highly diverse genus is still not well established. According to Dillon et al. (2001), *Sorghum* consists of 25 species distributed across Australia, the Pacific Islands, Southeast, East and South Asia, and much of Africa (**Table 1**). The USDA recently accepted one additional species to the genus—*Sorghum trichocladum* (Rupr. ex Hack.) Kuntze, which is native to Mexico, Guatemala, and Honduras (USDA, 2020). This species can be found only in limited locations (Spangler, 2003) and limited information is available on this species. Kew’s Angiosperm DNA C-values database, however, currently lists a total of 32 *Sorghum* species (Leitch et al., 2019). These differing classifications are based on diverse parameters, making sorghum taxonomy a complex and debatable area of study. In this review, *Sorghum* consists of 24 accepted species (USDA, 2020), with *S. bicolor* subspp. *verticilliflorum* and *drummondii* no longer considered separate species (**Figure 1**). The traditional classification of the genus, based on morphological parameters (Venkateswaran et al., 2014), is of limited value because it results in significant overlapping of the existing taxa. By contrast, recent studies of sorghum based on molecular evidence, such as phylogenetic analyses of DNA sequencing data, have been able to generate a classification with clear and precise groupings of these species (Venkateswaran et al., 2014). Although weak molecular evidence suggests that *S. trichocladum* is closely related to Australian taxa, the exact position of *S. trichocladum* in the phylogeny remains uncertain (Spangler, 2003).

**Table 1:** Taxonomic information, life form, and ploidy levels of taxa in the genus *Sorghum*.

Taxon and subgeneric section	Subgeneric section	Gene pool	Current Accepted taxonomy (USDA) (in AGG Grin Global database to be live Nov 2019)	Lifeform/Duration	Ploidy (2*n*)
*S.* ×*almum* Parodi	*Eu-sorghum*	Secondary	*S.* ×*almum* Parodi	Perennial	40
*S. arundinaceum* (Desv.) Stapf	*Eu-sorghum*	Primary	*Sorghum bicolor *subsp.* verticilliflorum *(Steud.) de Wet ex Wiersema & J. Dahlb.	Annual	20
*S. bicolor* (L.) Moench	*Eu-sorghum*	Primary	*S. bicolor* (L.) Moench	Annual	20
*S.* ×*drummondii* (Steud.) Millsp. & Chase	*Eu-sorghum*	Primary	*Sorghum bicolor *nothosubsp.* drummondii *(Steud.) de Wet ex Davidse	Annual	20
*S. halepense* (L.) Pers.	*Eu-sorghum*	Secondary	*S. halepense* (L.) Pers.	Perennial	40
*S. propinquum* (Kunth) Hitchc.	*Eu-sorghum*	Primary	*S. propinquum* (Kunth) Hitchc.	Perennial	20
*S. grande* Lazarides	*Parasorghum*	Tertiary	*S. grande* Lazarides	Perennial	30, 40
*S. leiocladum* (Hack.) C. E. Hubb.	*Parasorghum*	Tertiary	*S. leiocladum* (Hack.) C. E. Hubb.	Perennial	10, 20
*S. matarankense* E. D. Garber & Snyder	*Parasorghum*	Tertiary	*S. matarankense* E. D. Garber & Snyder	Perennial	10
*S. nitidum* (Vahl) Pers.	*Parasorghum*	Tertiary	*S. nitidum* (Vahl) Pers.	Perennial	10, 20
*S. purpureosericeum* (Hochst. ex. A. Rich.) Asch. & Schweinf.	*Parasorghum*	Tertiary	S*. purpureosericeum* (Hochst. ex. A. Rich.) Asch. & Schweinf.	Annual	10
*S. timorense* (Kunth) Buse	*Parasorghum*	Tertiary	*S. timorense* (Kunth) Buse	Perennial	10, 20
*S. versicolor* Andersson	*Parasorghum*	Tertiary	*S. versicolor* Andersson	Annual	10, 20
*S. amplum* Lazarides	*Stiposorghum*	Tertiary	*S. amplum* Lazarides	Annual	10, 30
*S. angustum* S. T. Blake	*Stiposorghum*	Tertiary	*S. angustum* S. T. Blake	Annual	10
*S. brachypodum* Lazarides	*Stiposorghum*	Tertiary	*S. brachypodum* Lazarides	Annual	10
*S. bulbosum* Lazarides	*Stiposorghum*	Tertiary	*S. bulbosum* Lazarides	Annual	10
*S. ecarinatum* Lazarides	*Stiposorghum*	Tertiary	*S. ecarinatum* Lazarides	Annual	10
*S. exstans* Lazarides	*Stiposorghum*	Tertiary	*S. exstans* Lazarides	Annual	10
*S. interjectum* Lazarides	*Stiposorghum*	Tertiary	*S. interjectum* Lazarides	Annual/Perennial	30
*S. intrans* F. Muell. ex Benth.	*Stiposorghum*	Tertiary	*S. intrans* F. Muell. ex Benth.	Annual	10
*S. plumosum* (R. Br.) P. Beauv.	*Stiposorghum*	Tertiary	*S. plumosum* (R. Br.) P. Beauv.	Annual	10, 20, 30, 40
*S. stipoideum* (Ewart & Jean White) C. A. Gardner & C. E. Hubb.	*Stiposorghum*	Tertiary	*S. stipoideum* (Ewart & Jean White) C. A. Gardner & C. E. Hubb.	Annual	10
*S. laxiflorum* F. M. Bailey	*Heterosorghum*	Tertiary	*S. laxiflorum* F. M. Bailey	Annual	40
*S. macrospermum* E. D. Garber	*Chaetosorghum*	Tertiary	*S. macrospermum* E. D. Garber	Annual	40
*S. trichocladum *(Rupr. ex Hack.) Kuntze	–	Tertiary	*S. trichocladum *(Rupr. ex Hack.) Kuntze	Perennial	–

### Eu-sorghum

*Eu-sorghum* is one of two major sections in the genus *Sorghum*. It is mainly distributed in Africa and southern Asia (Price et al., 2005a). In the original classification by Snowden (1955) *Eu-sorghum* was divided into two sub-sections, *Arundinaceae* and *Halepensia*. The sub-section *Arundinaceae* was further divided into two series, *Spontanea* (grass sorghum) and *Sativa* (grain sorghum). *Spontanea* contained 10 wild species whereas *Sativa* contained 31 cultivated species. Sub-section *Halepensia* was comprised of four wild rhizomatous taxa (De Wet and Harlan, 1971). Subsequently this classification was modified by many scientists. The number of members included in each group varied with the classification. For instance, the classification of de Wet et al. (1970) placed 17 wild species in the complex of *Spontanea*, while 31 cultivated species were in *Sativa* and the sub-section *Halepensia* contained four wild grass species.

In the currently accepted classification, *Eu-sorghum* is considered the “true sorghum” and contains three species, *S. bicolor*, *S. propinquum*, *S. halepense* and a hybrid species called, *Sorghum* ×*almum* Parodi (USDA, 2020).

*Sorghum bicolor* includes most cultivated sorghum lines, and is distinguished from other species by the bulky, open inflorescence and the non-pendulous branches separating at the base. *Sorghum bicolor* can be separated into three subspecies: subsp. *bicolor* (all cultivated sorghums), subsp. *verticilliflorum* (wild progenitors of cultivated sorghums), and nothosubsp. *drummondii* (Steud.) de Wet ex Davidse (weedy hybrids and the derivatives of hybridization between *S. bicolor* subspp. *bicolor* and *verticilliflorum*). The subsp. *verticilliflorum* (formerly known as *arundinaceum*) (Venkateswaran et al., 2019a) consists of four races of wild progenitors: *aethiopicum*, *arundinaceum*, *verticilliflorum*, and *virgatum*. The race *arundinaceum* is distributed mostly in Africa and has a large and exposed inflorescence as well as flexuous branches which are not dividing at the base. The desert grass, race *aethiopicum*, is widely distributed in the African Sahel and has a comparatively small, constricted inflorescence together with divided sub-erect branches. The race *virgatum*, characterized by a slender inflorescence and narrow, linear leaf blades, is widespread in north eastern Africa. The race *verticilliflorum* which is native to Africa and distributed in Madagascar can be characterized by a large open inflorescences with spreading branches divided at the base (Venkateswaran et al., 2014; Venkateswaran et al., 2019b). In the Snowden (1955) classification, there are seven weedy taxa recognized in nothosubsp. *drummondii* (also known as “Sudan grass”), which are commonly cultivated as forage. The currently accepted five races of subsp. *bicolor* are: *bicolor*, *guinea*, *kafir*, *caudatum*, and *durra*, which are categorized in this subspecies based only on their spikelet morphology with 10 intermediate races (Lazarides et al., 1991). Based on molecular evidence, Mace et al. (2013) also separated *guinea-margaritiferum* as a distinct race of subsp. *bicolor*, with this group previously being included in the broader *guinea* race. *Guinea-margaritiferums* represent an intermediate race between the wild subsp. *verticilliflorum* and the other domesticated races of subsp. *bicolor*.

*Sorghum propinquum* is a diploid rhizomatous wild perennial species that is distributed in Southeast Asia and Indian subcontinent. Smaller spikelets are distinctive features of *S. propinquum*. Another perennial species, *S. halepense*, also known as “Johnson grass,” is a tetraploid rhizomatous wild relative that is widespread in Southern Eurasia and India. According to Snowden classification 1955, this species contains members of three former species known as *S. halepense*, *S. miliaceum* (Roxb.) Snowden, and *S. controversum* (Steud.) (Venkateswaran et al., 2014). *Sorghum halepense* has comparatively large inflorescences than other two species. These two wild perennial species have given rise to hybrids and hybrid derivatives as a result of their introgression with *S. bicolor* (Dahlberg, 2000; Venkateswaran et al., 2014). *Sorghum ×almum*, for example, is a hybrid between *S. bicolor* and *S. halepense* (Duvall and Doebley, 1990; Dillon et al., 2007b).

### Parasorghum

The section *Parasorghum* includes seven species: *Sorghum grande* Lazarides, *Sorghum leiocladum* (Hack.) C. E. Hubb., *Sorghum matarankense* E. D. Garber & Snyder, *Sorghum nitidum* (Vahl) Pers., *Sorghum purpureosericeum* (Hochst. ex A. Rich.) Schweinf. & Asch., *Sorghum timorense* (Kunth) Büse, and *Sorghum versicolor* Andersson. Excepting *S. purpureosericeum* and *S. versicolor*, all *Parasorghum* species are native to Australia, with *S. grande*, *S. leiocladum* and *S. matarankense* all being endemic (Lazarides et al., 1991). *Sorghum grande* is a perennial diploid with a chromosome number of 30 or 40 (2n = 30, 40), distributed in the Northern Territory (isolated in Katherine region) and Queensland, Australia. *Sorghum nitidum* is also a perennial diploid with 2n = 10, 20 and is found in Queensland, New Guinea, and Southeast and East Asia. *Sorghum leiocladum* is a perennial with 2n = 20 which is distributed in southern Queensland, New South Wales, and northern Victoria. *Sorghum matarankense* is an annual species with 2n = 10 and it can be commonly seen in north-central parts of the Northern Territory, Australia. Likewise, *S. timorense* is an annual species with 2n = 10, 20, found in northern Australia and Timor. *Sorghum timorense* is distinguished by a minute, sessile spikelet with an obovoid caryopsis and a developed pedicellate spikelet. *Sorghum purpureosericeum* is an annual with chromosome number 2n = 10 and 20, and is found in India, the Sahel, and east and west tropical Africa. *Sorghum versicolor* is also annual, with a chromosome number of 2n = 10 and 20 and is found in eastern and southern Africa.

### Stiposorghum

The subgenus Stiposorghum contains a total of 10 species: Sorghum amplum Lazarides, Sorghum brachypodum Lazarides, Sorghum angustum S. T. Blake, Sorghum intrans F. Muell. ex Benth., Sorghum ecarinatum Lazarides, Sorghum bulbosum Lazarides, Sorghum plumosum (R. Br.) P. Beauv., Sorghum stipoideum (Ewart & Jean White) C. A. Gardner & C. E. Hubb, Sorghum interjectum Lazarides, and Sorghum exstans Lazarides, all of which are endemic to Australia. Among these, S. interjectum and S. plumosum are perennial species with 2n = 30, 40 and 2n = 10, 20, 30 respectively and the rest are annual species with 2n = 10. Interestingly, S. ecarinatum, S. bulbosum, S. plumosum, S. stipoideum, S. interjectum, and S. ecarinatum are distributed in both the Northern Territory and Western Australia, whereas S. amplum and S. brachypodum can only be found in Western Australia and the Northern Territory respectively. Sorghum intrans is found in north-western Northern Territory and S. exstans is found on Melville Island and adjoining mainland. These species have small sessile spikelet and a well-developed pedicellate spikelet (Lazarides et al., 1991).

### Heterosorghum

*Sorghum laxiflorum* F. M. Bailey is the sole member of *Heterosorghum*, and is native to Australia and New Guinea (Price et al., 2005a). In Australia, it is commonly found in Northern Territory and Queensland. It is an annual 2n = 40 plant with a comparatively large, sessile spikelet, obovoid to ellipsoid caryopsis and reduced spikelets (Lazarides et al., 1991).

### Chaetosorghum

*Sorghum macrospermum* E. D. Garber is the sole member of *Chaetosorghum* and is endemic to the Northern Territory (isolated to limestone outcrops around Katherine) (Price et al., 2005a). It is an annual, 2n = 40 species and has a small, sessile spikelet with an ovoid to ellipsoid caryopsis as well as a reduced pedicellate spikelet (Lazarides et al., 1991).

## Phylogenetic Relationships of the Genus *Sorghum*

The phylogenetic relationships within the genus *Sorghum* are complex, with several unresolved and potentially controversial issues. The primary gene pool (GP-1) of sorghum contains the cultivated species, *S.* *bicolor* and the wild species *S. propinquum* (Harlan and de Wet, 1971). The remaining members of *Eu*-*sorghum*, *S. halepense* and *S.*×*almum*, belong to the small secondary gene pool (GP-2) (Stenhouse et al., 1997; Dillon et al., 2001). Sorghum has a comparatively larger tertiary gene pool (GP-3) which includes all the species in the other four subgenera. Members of GP-1 and GP-2 are closely related to each other whereas the members of GP-3 are more distantly related (Harlan and de Wet, 1971). GP-3 species potentially contain many important genetic resources for sorghum improvement. However, species of this gene pool have been poorly studied as they are restricted to specific geographical areas (Bhattacharya et al., 2011).

The availability of the *S. bicolor* genome (Paterson et al., 2009) has facilitated phylogenetic studies of sorghum species based upon molecular analysis. To date, there have been several studies into the sorghum phylogeny based on nuclear genomic information together with some chloroplast genomic data. In a study by Sun et al. (1994) the *ITS* region of 13 sorghum species were sequenced covering all the subgenera, revealing the very close relationships within *Eu-sorghum*. *Sorghum bicolor* was found to be more closely related to *S. nitidum.* However, a similar study by Spangler et al. (1999), sequencing the *ndhF* gene of 39 species of the Tribe Andropogonoeae, suggested some contrary relationships within the genus as a whole. For example, they suggested a distant relationship of *S. nitidum* with *S. bicolor* by being closely related to *S. laxiflorum*, and many other opposite relationships compared to the results of Sun et al. (1994) (Spangler et al., 1999).

In an attempt to clarify these contradictory classifications, Spangler (2003), presented a revised unranked classification for the genus *Sorghum* based on molecular and morphological evidence. According to this classification, *Sorghum* can be divided into three genera namely, genus *Sorghum*, genus *Vacoparis* and genus *Sarga*. Although the relationships within these three genera are still unknown, some of the changes have been already accepted by International Code of Botanical Nomenclature (ICBN). The genus *Sorghum* in this classification contains three species (*Sorghum bicolor*, *Sorghum halepense*, and *Sorghum* *nitidum)*, the genus *Vacoparis* contains two species (*Vacoparis macrospermum* and *Vacoparis laxiflorum*), and the remaining genus *Sarga*, comprises eight species; *Sarga angustum*, S*arga intrans*, *Sarga leiocladum*, *Sarga plumosum*, *Sarga purpureosericeum*, *Sarga timorense*, *Sarga trichocladum*, and *Sarga versicolor*, which was created by collapsing sixteen species of sorghum into eight species.

Conversely, a more recent study (Dillon et al., 2007b), used 25 *Sorghum* taxa (not including *S. trichocladum*) to resolve the complex phylogeny of the *Sorghum* genus as many of the previous studies have resulted in contradictory classifications (Sun et al., 1994; Spangler et al., 1999; Dillon et al., 2001; Spangler, 2003; Dillon et al., 2004). Using a combined molecular analysis of *ITS1*, *ndhF*, and *Adh1*, all the sorghum species were placed in a monophyletic clade with two distinct lineages. The subgenus *Eu-sorghum* was in the same clade as *Chaetosorghum* and *Heterosorghum*, consistent with the close relationship of these two later subgenera to cultivated sorghum that was reported in earlier studies (Spangler et al., 1999; Dillon et al., 2001; Spangler, 2003; Dillon et al., 2004) and was later proved by a study by Ng’uni et al. (2010). The very close relationship of these two subgenera has been found in many other studies using morphological, cytogenetic and molecular studies despite their being considered as two separate subgenera. In addition, another clearly discrete clade was observed with all the *Parasorghum* and *Stiposorghum* species with three different clusters including *S. brachypodum* and *S. matarankense* in one cluster, *S. interjectum* and *S. ecarinatum* in another cluster, and *S. exstans*, *S. intrans* and *S. angustum* in the third cluster. The rest of the seven species in those two subgenera formed an unresolved polytomy within this clade with no clear separation for these species. Most importantly, this study demonstrates that most of the modifications in the revised classification of Spangler (2003) are not valid except for placing *Chaetosorghum* and *Heterosorghum* together in one section. Clearly more molecular evidence is required before reclassifying the genus *Sorghum* into three subgenera.

An alternative explanation for the confusion around the *Sorghum* taxonomy is the possibility that the genus is polyphyletic within the tribe Andropogoneae (Hawkins et al., 2015). Hawkins et al. (2015) compared four nuclear loci data in 16 sorghum species together with 57 species in Andropogoneae and were able to identify two major lineages; clade I: *Eu-sorghum, Chaetosorghum* and *Heterosorghum*, and clade II; *Stiposorghum* and *Parasorghum* supporting previous studies done by Duvall and Doebley (1990); Sun et al. (1994), and Dillon et al. (2001). These studies were able to provide evidence of the sister relationships of these species to *Eu-sorghum* that was contrary to the single genus *Vacoparis* proposed by Spangler (2003). In clade II of the study of Hawkins et al., 2015, *S. matarankense* (*Parasorghum*) is resolved within *Stiposorghum* suggesting that it might belong to *Stiposorghum* or *Parasorghum* might be paraphyletic. However, the relationships within the clade *Stiposorghum* were only supported by low bootstrap values making them more difficult to resolve.

## Gene Flow Between Wild and Cultivated Sorghum

It has been found that many major crops are capable of natural hybridization with their wild relatives (Ellstrand et al., 1999) due to the fact that they are biologically in the same genus as their wild progenitors (Harlan and de Wet, 1971). The introgression of genes from wild relatives into crops supports the increasing genetic diversity of many species (Arnold, 2004). It is well known that diversity of wild progenitors is usually higher than that of the corresponding cultivated varieties. This is a result of domestication in which the bottleneck effect has limited the genetic diversity (Papa et al., 2005). Thus, the wild relatives of the crops may harbor valuable genetic resources and unique sources of diversity. Many studies have been carried out to study the extent and direction of the gene flow in crop-wild population complexes such as maize (Hufford et al., 2013), barley (Jakob et al., 2014), and rye (Schreiber et al., 2019), but studies on sorghum are limited. *Sorghum* *bicolor* subsp. *bicolor* has the advantage of having a wild progenitor, subsp. *verticilliflorum*, and its weedy relative, *S. drummondii*, which are interfertile with the cultivated species, and also grow sympatrically with cultivated forms (de Wet et al., 1970; de Wet, 1978). Studies have been done to detect the direction of gene flow through the cultivated, wild and weedy forms of sorghum, mainly based on the agricultural regions in Kenya (Mutegi et al., 2010; Mutegi et al., 2012), Ethiopia and Niger (Tesso et al., 2008), northern Cameroon (Barnaud et al., 2009), and western Africa (Sagnard et al., 2011). All of these studies have had the same conclusion, suggesting that the crop-to-wild gene flow is more common. The studies have also emphasized a close genetic relationship between wild and crop species of sorghum.

Mutegi et al. (2012) concluded that gene flow is asymmetric by proving the rate of gene flow from crop-to-wild is higher than the gene flow from wild-to-crop, and also proposed three scenarios that could affect this asymmetric gene flow. Firstly, the sizes of the crop and wild populations might be a reason for this asymmetric gene flow which favors the larger population size of the crops compared to the smaller size of the wild populations in most agricultural lands in Africa. Farmers tend to remove wild progenitors of sorghum, considering them to be weeds. As a result, the cultivated sorghum plants produce more pollen than the wild sorghum, resulted in higher rates of pollen flow from crop to wild. Secondly, differences in the mating systems between cultivated and wild sorghum species could be a contributing factor. The higher rates of outcrossing in wild sorghums relative to cultivated sorghums facilitate the cross pollination. Thirdly, seed selection by farmers has an effect on the asymmetric gene flow. According to this concept, farmers selecting against early generations of hybrids can reduce the possibility of gene introgression from wild plants to cultivated plants. The gene flow between cultivated and wild forms has played a key role in producing intermediate species of sorghum. Doggett (1965), suggested that the balance between natural selection for wild traits and farmer selection for cultivated traits resulted in the great genetic diversity of sorghum.

## Current Issues With Sorghum

### Genetic Bottlenecks

The wild ancestors of sorghum have various advantageous traits such as palatable grains, high yield, wide distribution, and higher abundance over large areas. As a result, they became the main food source of early people in African savannah (de Wet and Shechter, 1977). However, with the process of domestication, most of these morphological traits were changed due to automatic selection. Tillering of the plants as well as aerial branching were reduced to have plants with only one main stem with a single inflorescence which ultimately resulted in uniform maturity. An extremely compact inflorescence was produced by contracting the axis and branches. The grain size became larger as a result of an increase in the amount of endosperm and subsequently the shape of the grain changed from elliptic to become more obovate. The breakable spikelet clusters changed to one remaining attached to the rachises at maturity (Venkateswaran et al., 2019a).

Many studies supported the concept of co-existence of wild sorghum with the cultivated sorghum in many agricultural fields of Africa (Barnaud et al., 2009; Mutegi et al., 2010; Mutegi et al., 2012). Using pure cultivated, pure wild and putative hybrids, they have proven that there is a clear genetic divergence between the populations of pure wild and pure cultivated. Interestingly, the putative hybrid group played an important role in terms of genetic diversity by having an intermediate position in between the pure wild and pure cultivated populations. Genetic diversity reduction is known to be a result of domestication. According to the study of Mutegi et al. (2011), the genetic diversity of wild sorghum is significantly higher than the genetic diversity of cultivated sorghum in Kenya. These results agreed with the results of similar studies of Barnaud et al. (2009) and Sagnard et al. (2011) which were carried out at a local scale in Cameroon and national scale in Mali and Guinea respectively. In contrast, a parallel study carried out on a local scale by Mutegi et al. (2012) indicated that the genetic diversity between these two groups were similar in terms of gene diversity, allelic richness, and private allelic richness. However, they were able to discover 19 unique alleles in cultivated sorghum and 31 unique alleles in wild sorghums suggesting that the two gene pools were able to preserve their genetic diversity to some extent even if they were subjected to gene flow. These rare alleles of the wild plants might be linked with the traits such as drought tolerance or disease resistance.

A more recent study of Fernandez et al. (2014) has assessed the genetic diversity of landraces and wild/weedy relatives of sorghum in western Kenya using SSR markers. These authors have concluded that wild sorghum populations harbor a higher genetic diversity relative to the cultivated forms. Furthermore, in the cluster analysis although the cultivated and wild forms formed separate groups, the weedy hybrids failed to have a separate cluster from the wild forms suggesting that so called “hybrids” are closely related to the wild sorghums. Fernandez et al. (2014) outlined several reasons for this reduced gene flow and genetic diversity. For instance; farmer selection for desired traits and agronomic practices such as weeding have limited the gene flow and diversity within the cultivated species by means of reducing the cross pollination between wild and cultivated sorghums (Okeno et al., 2012). There might be several reasons for these controversial conclusions of genetic diversity differences between the wild and cultivated populations of sorghums. Differences in the experimental design, experimental area, sample size, and number of markers can affect the results of these studies. Therefore, a broader scale study which covers almost all the regions and species of sorghum is required for further validation these concepts.

Although many studies have indicated that the genetic diversity of sorghum has been reduced due to domestication, the study of Venkateswaran et al. (2019a) claimed that the variability of the plant species within the group has increased with domestication. Authors have stated that the variability in plant types, spikelet types, grain types, and inflorescence types as well as the distribution of the species have been greatly increased with the process of domestication. The morphological changes associated with domestication often gave rise to adaptations to new environments which enhanced the range of the species. These new characteristics were fixed to the new group of cultivated sorghum plants.

**Figure 2:**
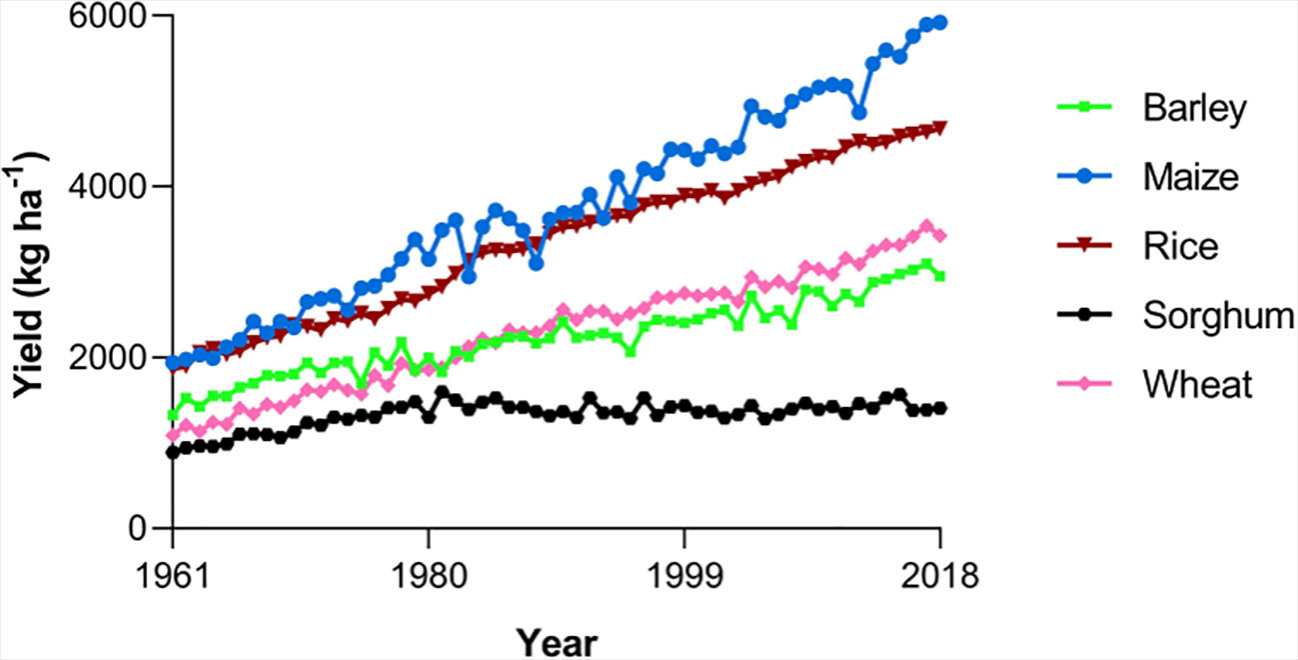
Trends in the total yields of the world’s five most important cereal crops. Data obtained from FAO (2019).

Grain sorghum farmers have been facing difficulty in attempts to increase yields per unit of land. While most other major cereal crops have shown significant improvement in yield gains in the past 50 years, sorghum yields have plateaued, with levels peaking in 1981 (**Figure 2**) (Mason et al., 2008; Aruna and Cheruku, 2019). One potential cause of this plateau is a low rate of genetic enhancement, with breeding programs for other crops generally receiving more funding than sorghum during this time, and consequently being more successful (Frey, 1996; Mason et al., 2008). This is problematic due to the unlikeliness of future increases in sorghum production through a greater availability of farmland, meaning the majority of increases must come from further intensification of farming. Successful yield gains through breeding have sometimes led to losses in other crop traits, such as various Indian sorghum lines being created and used specifically for high yields despite reductions in grain quality (Aruna and Cheruku, 2019). We must continue to tackle sorghum’s genetic homogeneity issues, increasing the amount of research done and the breadth of methods used, in order to increase yields again without sacrificing nutrition.

## Crop Wild Relatives in Sorghum Improvement

### Barriers to Use of Sorghum’s Wild Relatives

Undomesticated sorghum species harbor beneficial traits which can be employed as prospective markers to the phylogenetic relationships within the genus as well as between similar plant families. One of the major constraints to utilizing these genetic resources of wild relatives is the barriers to gene transfer between cultivated crops and their wild relatives (Bevan et al., 2017). Some of the sorghum species in the primary and secondary gene pools have been extensively used in genetic studies since they have few genetic incompatibilities with *S. bicolor*. However, most of the wild sorghum species belong to the tertiary gene pool and gene transfer to the cultivated sorghum species is difficult. Recent phylogenetic studies have revealed the two undomesticated species *S. laxiflorum* and *S. macrospermum* as the most closely related species to the cultivated sorghum species (Dillon et al., 2007c). Many unsuccessful attempts have been made to produce viable hybrids (Garber and Snyder, 1951; Sun et al., 1994; Huelgas et al., 1996). Gene transfer from the crop wild relatives to the cultivated sorghum species is challenging for several reasons. The main reason is the strong pre- and post-zygotic reproductive barriers between wild and domesticated species. These sterility barriers can be seen as a result of differences in genome size, chromosome morphology, pollen-pistil incompatibilities, and embryo abortions (Garber and Snyder, 1951; Price et al., 2005b). Hybrid embryo formation may be impossible due to the pollen-pistil incompatibilities between *S. bicolor* and wild species (Hodnett et al., 2005). However, successful efforts of hybridization have been reported with artificial hybridization techniques such as embryo rescue (Price et al., 2005b). One successful attempt has been reported between the species *S. bicolor* and *S. macrospermum*, using embryo rescue methods (Price et al., 2005b). Techniques such as the use of bridge species, irradiation of pollen grains, and chromosome doubling have also been used to overcome these sterility barriers (Kumari et al., 2016). Kuhlman et al. (2010) have also successfully developed a *S. bicolor* line which is homozygous for the recessive *iap* (inhibition of alien pollen) gene, allowing pollen tubes to grow to completion, even when the pollen is from a GP-3 species. Hybrids have since been made by crossing *S. bicolor* with *S. macrospermum* (Kuhlman et al., 2010), and also with *Saccharum* spp. (Hodnett et al., 2010). A detailed account of attempts of producing hybrids between cultivated sorghum and wild sorghum has been explained in a review of Ohadi et al. (2017) (**Table 2**).

**Table 2:** Experimental details of hybridization between *S. bicolor* and its wild relatives (Ohadi et al., 2017).

Taxon	Status	References
*S. bicolor and S. almum*	Successful hybrids	Endrizzi, 1957
*S. bicolor* and *S. angustum*	Unsuccessful (*in vivo* rescue of the developing embryos were required)	Price et al., 2006
*S. bicolor* and *S. bicolor* nothosubsp*. drummondii*	Unassisted hybridization	Schmidt et al., 2013
*S. bicolor* and *S. bicolor* nothosubsp*. drummondii*	Successful hybrids	Werle et al., 2014
*S. bicolor* and *S. halepense*	Successful hybrids	Endrizzi, 1957; Hadley, 1958; Sangduen and Hanna, 1984; Piper and Kulakow, 1994; Cox et al., 2002; Dweikat, 2005; Magomere et al., 2015
*S. bicolor* and *S. halepense*	Natural introgression	Morrell et al., 2005
*S. bicolor* and *S. macrospermum*	Successful introgression using embryo rescue	Price et al., 2006; Kuhlman et al., 2010
*S. bicolor* and *S. nitidum*	Unsuccessful (*in vivo* rescue of the developing embryos were required)	Price et al., 2006
*S. bicolor* and *S. propinquum*	Successful hybrids but no use in sorghum improvement	Paterson et al., 1995; Wooten, 2001
*S. bicolor* and *S. versicolor*	Successful hybrids	Sun et al., 1991
*S. bicolor* and *S. bicolor* subsp. *verticilliflorum*	Successful hybrids	Cox et al., 1984; Jordan et al., 2004

Additionally, sorghum CWR may have been overlooked historically due to their apparent lack of usefulness regarding advantageous agricultural traits. This trend has been seen in many CWR (Jansky et al., 2013), including wild sorghum species having been overlooked in the past due to their low yields and “weedy” characteristics (Cox et al., 1984; McWhorter, 1989). However, there are various reasons why *Sorghum* species should no longer be viewed this way. Several of these species have been shown to possess traits which would be desirable in sorghum crops (Kamala et al., 2002; Venkateswaran, 2003; Cowan et al., 2020). Increasing genetic heterogeneity through hybridization can also be unexpectedly beneficial through heterosis—enhancement of traits through mixing genes of two genetically-distinct parents. Some benefits can be phenotypically obvious, for example with Jordan et al. (2004) finding some hybrids of *S. bicolor* subspp. *bicolor* and *verticilliflorum* with higher yields than either of the parent plants. This finding was surprising given that subsp. *verticilliflorum* typically has low grain yields. Other benefits of heterosis might be less immediately noticeable, including reduced susceptibility to pests, pathogens, and environmental changes (Chen, 2010).

### Use of Gene Pools 1 and 2

Due to the incompatibly of crossing *S. bicolor* with species in GP-3, most existing hybrids have been made through crosses of *S. bicolor* with members of gene pools 1 and 2 (Duncan et al., 1991). These include: *S. bicolor* subsp. *verticilliflorum* (Cox et al., 1984; Jordan et al., 2004) and *S. propinquum* (Wooten, 2001) being used to increase yield; *S. halepense* being used to introduce perennialism (Cox et al., 2002; Dweikat, 2005); and *S. propinquum* being used to increase height and earliness of development (Wooten, 2001) (**Table 3**). There have also been countless crosses between different commercial lines of the crop (Rosenow and Clark, 1982; Duncan et al., 1991; Aruna and Cheruku, 2019).

**Table 3:** Details of potential wild sorghum species which can be used to improve cultivated sorghum.

Taxon	Gene pool	Traits	Status	References
*S. propinquum*	1	Increase grain yield, increase height, and earliness of development	Successfully introgessed to *S. bicolor*	Wooten, 2001
*S. bicolor* subsp. *verticilliflorum*	1	Increase grain yield	Successfully introgessed to *S. bicolor*	Cox et al., 1984; Jordan et al., 2004
*S. halepense*	2	Perennialism	Successfully introgessed to *S. bicolor*	Cox et al., 2002; Dweikat, 2005
*S. bicolor* subsp. *verticilliflorum*	1	Ability to grow in drought conditions, seeds with tolerance to high temperatures, high yield, parasite resistance	Reported as potential candidates for sorghum improvement	Bramel-Cox and Cox, 1988; Rich et al., 2004
*S. bicolor* nothosubsp. *drummondii*	1	Allelopathic properties, resistance to ergot and nematodes	Reported as potential candidates for sorghum improvement	Mojtahedi et al., 1993; Tsukiboshi et al., 1998; Viaene and Abawi, 1998; Baerson et al., 2008
*S. halepense*	2	Resistance to green bug, chinch bug, and sorghum shoot fly	Reported as potential candidates for sorghum improvement	Nwanze et al., 1995; Dweikat, 2005
*S. angustum*	3	Resistance to egg laying by sorghum midge	Reported as potential candidates for sorghum improvement	Sharma and Franzmann, 2001
*S. amplum*	3	Resistance to egg laying by sorghum midge	Reported as potential candidates for sorghum improvement	Sharma and Franzmann, 2001
*S. bulbosum*	3	Resistance to egg laying by sorghum midge	Reported as potential candidates for sorghum improvement	Sharma and Franzmann, 2001
*S. macrospermum*	3	Insect and disease resistance, higher growth rate and an insignificant aboveground dhurrin content under drought conditions	Successfully introgessed to *S. bicolor*	Kuhlman et al., 2008; Cowan et al., 2020
*S. brachypodum*	3	Higher growth rate and an insignificant aboveground dhurrin content under drought conditions	Reported as potential candidates for sorghum improvement	Cowan et al., 2020
*S. exstans*	3	Resistance to shoot fly	Reported as potential candidates for sorghum improvement	Kamala et al., 2009
*S. stipoideum*	3	Resistance to shoot fly	Reported as potential candidates for sorghum improvement	Kamala et al., 2009
*S. matarankense*	3	Resistance to shoot fly	Reported as potential candidates for sorghum improvement	Kamala et al., 2009
*S. leiocladum*	3	Cold tolerance	Reported as potential candidates for sorghum improvement	Fiedler et al., 2016

There are also various other traits within gene pools 1 and 2 which have been listed as potentially useful for introgression into *S. bicolor*. Harlan (1992), reported that the wild race *arundinaceum* was adapted to growing in wet climates, an adaptation not common in cultivated sorghum species. The wild race *virgatum* can grow in drought conditions and their seeds have been shown to be tolerant to high temperatures (Bramel-Cox and Cox, 1988). In addition, Bramel-Cox and Cox (1988), showed that high yielding wild species *arundinaceum*, *virgatum*, and *verticilliflorum*, could be used to increase the yield of domesticated sorghum. These wild races also have resistance to the parasitic weed *Striga asiatica* Lour., a useful trait in sorghum cultivation (Rich et al., 2004). Other potentially useful traits in sorghum’s GP-1 and GP-2 include: *S. bicolor* nothosubsp. *drummondii*’s allelopathic properties, which reduce the growth of weeds in the cultivated field (Baerson et al., 2008), and resistance to ergot (Tsukiboshi et al., 1998) and nematodes (Mojtahedi et al., 1993; Viaene and Abawi, 1998); and *S. halepense*’s resistance to pests such as green bug, chinch bug, and sorghum shoot fly (Nwanze et al., 1995; Dweikat, 2005) (**Table 3**). Meanwhile, continued crosses between commercial lines will continue to contribute to recombination efforts, while also potentially generating serendipitous new phenotypes (such as yield gains) through heterosis.

### Use of Gene Pool 3

Although sorghum’s GP-3 has not yet extensively been used in crop improvement, it potentially contains a high level of genetic diversity for use in sorghum improvement. This diversity is suggested by the ability of these species to adapt to a range of edaphic conditions, with Australia’s native sorghums collectively covering diverse habitats including rocky slopes, sand dunes, grasslands, and forests (Lazarides et al., 1991). The niche diversity of GP-3 is much greater than that of GP-1 and GP-2, potentially providing genetic resources with which the environmental tolerances of sorghum crops could be expanded. For example, there has been great interest in increasing sorghum’s tolerance to cold temperatures in order to greatly expand the zone in which it can be grown (Fiedler et al., 2016, Yu and Tuinstra, 2001). *Sorghum leiocladum* could be a good candidate species for cold tolerance genes due to its presence in temperate regions of New South Wales and Victoria, Australia. Similarly, Cowan et al., 2020, found multiple GP-3 species with greater tolerance to drought than domesticated sorghum, including *S. brachypodum* and *S. macrospermum*. Further research into GP-3 could unveil more environments to which wild species could offer novel tolerance genes. Species across GP-3 have also been shown to be resistant to biotic stressors including sorghum shoot fly (Venkateswaran, 2003; Kamala et al., 2009), spotted stem borer (Venkateswaran, 2003), and downy mildew (Kamala et al., 2002), as well as *S. angustum*, *S. amplum* and *S. bulbosum* all showing resistance to egg laying by sorghum midge (Sharma and Franzmann, 2001) (**Table 3**). The identification of such traits despite the limited number of studies conducted on sorghum’s GP-3 suggests that there is high potential for finding further agronomically advantageous traits in this gene pool.

Cowan et al. (2020) also found that, in contrast to other cyanogenic crops, the leaf cyanogenic glucoside content of drought stressed wild sorghums is lower than that of the cultivated species (Cowan et al., 2020). Interestingly, findings of this study revealed that drought stress significantly increased the dhurrin concentration of the aboveground parts of *S. bicolor*, while the wild species were not significantly affected. Specifically, the two wild species *S. macrospermum* and *S. brachypodum* were able to maintain a higher growth rate and an insignificant aboveground dhurrin content. The regulation of the formation cyanogenic glucosides in wild sorghum species has not yet been studied in detail or compared to that of cultivated *S. bicolor*. Understanding the gene expression and regulation of cyanogenesis related genes in wild relatives of sorghum would be a crucial step in utilizing the useful traits in wild sorghum in crop improvement (Cowan et al., 2020).

### Priorities for Future Work

**Figure 3:**
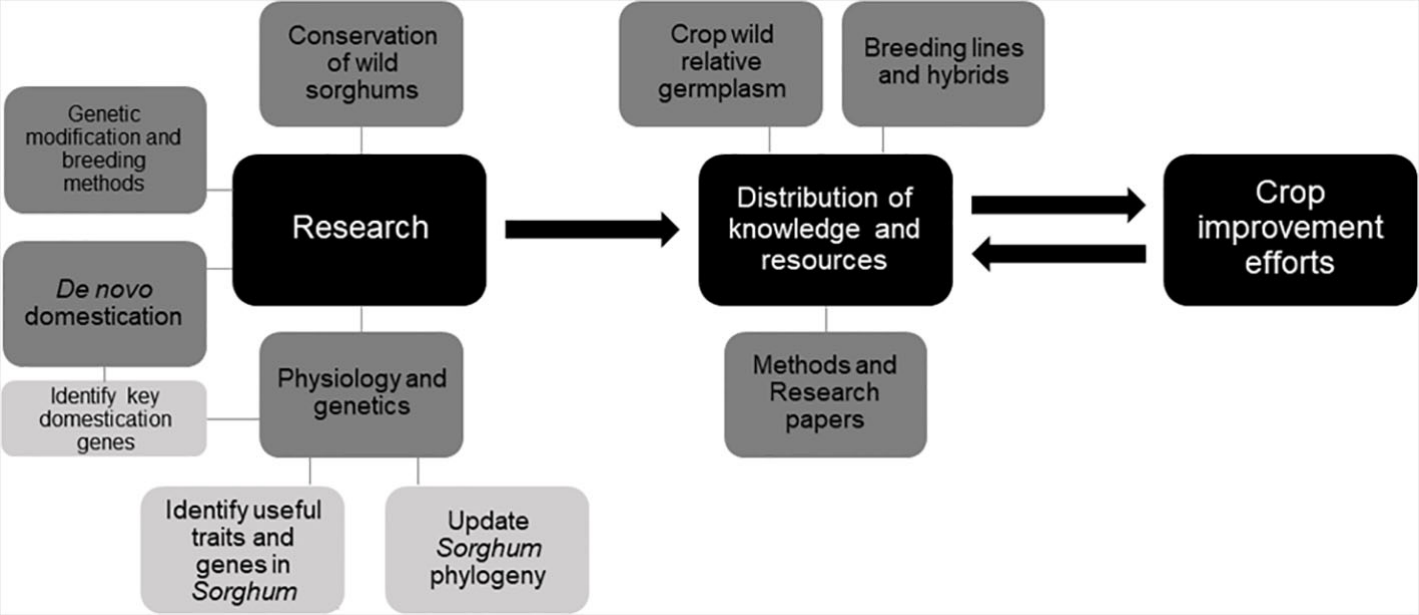
Roadmap towards the use of sorghum’s wild relatives in crop improvement.

In order to maximize the impact of sorghum improvement using CWR, various steps must be taken to improve how current work is executed. These steps include further development and distribution of *S. bicolor* lines which can interbreed with species outside GP-1 and GP-2, further improvements in the sorghum GM process, increased accessibility for crop developers and researchers to CWR germplasm, knowledge, and introgression technology, and a better understanding of how each of sorghum’s CWR might be valuable to the crop improvement process. *De novo* domestication of wild sorghums might also be a valuable method through which new sorghum lines could be developed (Fernie and Yan, 2019). Continued research into the morphology and physiology of CWR species will allow us to determine which species are potentially the most suitable as genetic sources for crop improvement, as well as for undergoing *de novo* domestication, taking into account potential uses, yields, and crop safety (e.g. storage of cyanogenic glucosides). Some of sorghum’s key domestication genes have already been identified (Meyer and Purugganan, 2013; Tao et al., 2017). Further elucidation of the *Sorghum* phylogeny might also help in the introgression process through allowing a better understanding of the relatedness of each species to the target crop (**Figure 3**).

All of these steps also rely on the continued conservation of the CWR species and the intraspecific genetic diversity within them. A combination of *ex situ* and *in situ* conservation techniques is vital for preserving the maximum genetic diversity (Engels et al., 2008). Currently, the world sorghum germplasm collection contains more than 200,000 accessions (FAO, 2009). Among these germplasm collections, ICRISAT (International Crops Research Institute for the Semi-Arid Tropics) has the world’s depository of sorghum germplasm collection, including many accessions from GP-1 and GP-2, as well as some GP-3 accessions (Wang et al., 2015). The main GP-3 germplasm collections are located in Australia at the Australian Grains Genebank (Bhattacharya et al., 2011; Genesys-pgr, 2020) with additional germplasm—mainly of the same lines as those held by the Australian Grains Genebank—held overseas by organizations such as the USDA Agricultural Research Service and the Millennium Seed Bank. Because most *Sorghum* species are native to Australia, *in situ* protections in the nation are vital for protecting the genus’ diversity. However, *in situ* protections of GP-3 species across Africa and Asia are also necessary, as these represent the genetic resources which are most easily crossed with the crop.

## Conclusion

Sorghum is an immensely valuable multipurpose crop with several end user products. The genus *Sorghum* is rich in diversity with a highly beneficial reservoir of untapped genetic resources, especially in the tertiary gene pool. The wild relatives of sorghum contain many expedient traits which can be utilized in crop improvement. However, exploitation of these extremely valuable traits in crop improvement is still hindered due to the limited availability of genetic information on these wild sorghum species. Furthermore, the genetic barriers in gene transfer between wild and cultivated sorghum species are challenging. However, with recent advances in next generation sequencing technologies, more genomic data will become available to researchers. This will extend the development of sorghum improvement programs using the rich, yet unexploited genetic resources in sorghum’s wild relatives. These resources may also support the development of new crops in the tribe Andropogoneae (Dillon et al., 2007c) either by introgression of useful genes into genera such as *Saccharum* or by domestication of further *Sorghum* species.

## Author Contributions

GA and HM led the writing of the manuscript. All authors contributed to the article and approved the submitted version.

## Funding

This work was funded by an Australian Research Council Discovery Project grant to RG and RH (DP180101011).

## Conflict of Interest

The authors declare that the research was conducted in the absence of any commercial or financial relationships that could be construed as a potential conflict of interest.
